# ZFP36L1 and ZFP36L2 inhibit cell proliferation in a cyclin D-dependent and p53-independent manner

**DOI:** 10.1038/s41598-018-21160-z

**Published:** 2018-02-09

**Authors:** Fat-Moon Suk, Chi-Ching Chang, Ren-Jye Lin, Shyr-Yi Lin, Shih-Chen Liu, Chia-Feng Jau, Yu-Chih Liang

**Affiliations:** 1 Division of Gastroenterology, Department of Internal Medicine, Wan Fang Hospital, Taipei Medical University, Taipei, Taiwan; 2 Division of Allergy, Immunology and Rheumatology, Department of Internal Medicine, School of Medicine, College of Medicine, Taipei Medical University, Taipei, Taiwan; 3 Division of Rheumatology, Immunology and Allergy, Taipei Medical University Hospital, Taipei, Taiwan; 4 School of Medical Laboratory Science and Biotechnology, College of Medical Science and Technology, Taipei Medical University, Taipei, Taiwan; 5 Department of Primary Care Medicine, Taipei Medical University Hospital, Taipei, Taiwan; 6 Department of General Medicine, School of Medicine, College of Medicine, Taipei Medical University, Taipei, Taiwan; 7 Traditional Herbal Medicine Research Center, Taipei Medical University Hospital, Taipei, Taiwan; 8 Ph.D. Program in Medical Biotechnology, College of Medical Science and Technology, Taipei Medical University, Taipei, Taiwan

**Keywords:** Cell growth, Cell division

## Abstract

ZFP36 family members include ZFP36, ZFP36L1, and ZFP36L2, which belong to CCCH-type zinc finger proteins with two tandem zinc finger (TZF) regions. Whether ZFP36L1 and ZFP36L2 have antiproliferative activities similar to that of ZFP36 is unclear. In this study, when ZFP36L1 or ZFP36L2 was overexpressed in T-REx-293 cells, cell proliferation was dramatically inhibited and the cell cycle was arrested at the G1 phase. The levels of cell-cycle-related proteins, including cyclin B, cyclin D, cyclin A, and p21, decreased; however, p53 increased in ZFP36L1-or ZFP36L2-overexpressing T-REx-293 cells. Forced expression of ZFP36L1 or ZFP36L2 also inhibited cell proliferation and cyclin D gene expression in three human colorectal cancer cell lines: HCT116 p53^+/+^, HCT116 p53^−/−^, and SW620 (mutated p53) cells. However, it increased p53 and p21 expression only in HCT116 p53^+/+^ cells. Knockdown of ZFP36L1 or ZFP36L2 increased cell proliferation and cyclin D expression; furthermore, the mutation of the TZF of ZFP36L1 or ZFP36L2 caused them to lose their antiproliferative ability, to the extent that they could not inhibit cyclin D expression in these three cell lines. The results indicated that ZFP36L1 and ZFP36L2 play a negative role in cell proliferation; the underlying mechanisms might be mediated through a cyclin D-dependent and p53-independent pathway.

## Introduction

Zinc finger proteins are the most abundant proteins in eukaryotic genes^1,2^ and the largest transcription factor family in the human genome^3^. According to their structure and function, zinc finger proteins can currently be roughly divided into 14 families. The CCCH-type zinc finger protein is one member of the family, which contains three cysteine and one histidine residue^4^. Unlike other zinc finger protein families, which are mostly defined as DNA- or protein-binding proteins, a CCCH-type zinc finger motif directly binds to RNA; therefore, CCCH-type zinc finger proteins are identified as RNA-binding proteins^5^. The ZFP36 protein family belongs to CCCH-type zinc finger proteins and has four members: ZFP36 (also called tristetraprolin, TIS11, TTP, NUP475, or GOS24), ZFP36L1 (also called TIS11b, Berg36, ERF1, or BRF1), ZFP36L2 (also called TISlld, ERF2, or BRF2), and ZFP36L3^6^. However, ZFP36L3 is not present in humans^7^. All three of the human proteins (ZFP36, ZFP36L1, and ZFP36L2) have two highly conserved TZF domains that are responsible for binding to the AU-rich elements (AREs) of certain messenger mRNAs, resulting in the instability and degradation of the mRNAs^8,9^.

In ZFP36-knockout mice, macrophages lacking ZFP36 display increased tumor necrosis factor (TNF)-α mRNA stability and TNF-α production^10^. Other studies found that ZFP36 family proteins negatively regulate the mRNA stability of granulocyte macrophage colony-stimulating factor (GM-CSF)^11^, vascular endothelial growth factor (VEGF)^12,13^, cyclooxygenase (COX)-2^14^, cyclin D^15^, c-Myc^15^, and bcl-2^16,17^;. Therefore, the functions of the ZFP36 family are linked to the regulation of inflammation, apoptosis, proliferation, and angiogenesis^18^. Notably, the ZFP36 protein family also binds to the 3′ untranslated region (UTR) on its own mRNA and negatively regulates its expression^19,20^. ZFP36 promotes destabilization of interleukin (IL)-8 and IL-10 mRNA through deadenylation^21,22^, and decreases the level of GM-CSF mRNA by shortening the poly A tail of GM-CSF mRNA^11^. In addition, ZFP36 and ZFP36L1 were found to interact with RNA degradation components, including decapping subunits (DCP1 and DCP2), 5′→3′ exoribonuclease, deadenylase, and the exosome complex component RRP4^23^. Studies have shown that ZFP36 also interacts with other proteins that are not directly related to mRNA degradation. ZFP36 associates with the nuclear pore protein Nup214 in an interaction that regulates ZFP36 localization^24^. ZFP36 also binds directly to the retroviral Tax oncoprotein and acts as a transcriptional regulator of viral gene expression^25^.

ZFP36, ZFP36L1, and ZFP36L2 are widely expressed in the early stages of lymphocyte development, playing critical roles in controlling the expression of several cyclins and cyclin-dependent kinases (Cdks), as well as cell proliferation^26^. Double conditional knockout of ZFP36L1 and ZFP36L2 upregulates the expression of cyclin D1 and cyclin D3 during B cell development^27^. Using individual nucleotide crosslinking and immunoprecipitation (iCLIP), ZFP36L1 was discovered to be able to bind to AREs in the 3′UTRs of a group of mRNAs that encode cell cycle regulators^27^. Therefore, ZFP36L1 can be considered an RNA regulon^26^. Deficiencies in ZFP36L1 and ZFP36L2 significantly increased cell proliferation, as well as increasing cell cycle regulators, such as cyclin D3 and cyclin E2 in mice CD4(−) CD8(−) double negative thymocytes^28^. Moreover, thymocyte proliferation and development was inhibited in GFPZFP36L1 transgenic mice. ZFP36L1 also downregulated Cdk6 expression by binding to the AREs of Cdk6 mRNA 3′UTR and blocked the monocyte/macrophage differentiation CD34(+) hematopoietic stem/progenitor cells^29^. Senescent fibroblasts secrete a group of factors collectively termed the senescence-associated secretory phenotype (SASP), which can promote the epithelial-to-mesenchymal transition of epithelial cancer cells and enhance the tumorigenic potential of cancer cells^30^. ZFP36L1 was discovered to directly decay SASP components in an ARE-dependent manner. However, the phosphorylation of ZFP36L1 by MAPKAPK2 results in the inhibition of ZFP36L1^30^. By contrast, ZFP36L1 plays a positive role in marginal zone B cell identity and survival through limiting several gene expressions, including IRF8 and KLF2^31^.

Numerous studies have shown that the ZFP36 protein family is associated with antitumor and anti-inflammatory activities. ZFP36 is a crucial negative regulator of TNF-α as well as other proinflammatory cytokines; a reduction in ZFP36 expression contributes to the development of immune-related diseases, including rheumatoid arthritis, systemic lupus erythematosus, and ulcerative colitis^5^. Overexpression of ZFP36 inhibits gene expressions of FOS, EGR1, and TNF-α, which are critical in macrophage differentiation and atherosclerosis^32^. Notably, Myc can bind to the initiator (Inr) elements of ZFP36 and ZFP36L1; this binding is associated with their reduced transcription^33^. However, ZFP36 (but not ZFP36L1) impairs Myc-induced lymphomagenesis. In addition, ZFP36 is a prognostic indicator of breast cancer^34^. In lung cancer cells, overexpression of ZFP36 decreased the expression of LATS2 mRNA, a putative tumor suppressor gene^35^. Overexpression of the ZFP36 protein family can also induce apoptosis in a variety of cells, including HeLa (human cervical cancer cells), U2OS (osteosarcoma cells), SAOS2 (osteosarcoma cells), and 3T3 (mouse fibroblast cells)^36,37^.

ZFP36 protein has numerous biological functions. However, the biological functions of ZFP36L1 and ZFP36L2 and their underlying molecular mechanisms remain unclear. In this study, we investigated the roles of ZFP36L1 and ZFP36L2 in regulating cell proliferation and related gene expressions by using overexpression and knockdown techniques, and TZF mutants of ZFP36L1 and ZFP36L2.

## Results

### ZFP36L1 and ZFP36L2 inhibited cell proliferation but did not cause the death of T-REx-293 cells

**Figure 1:**
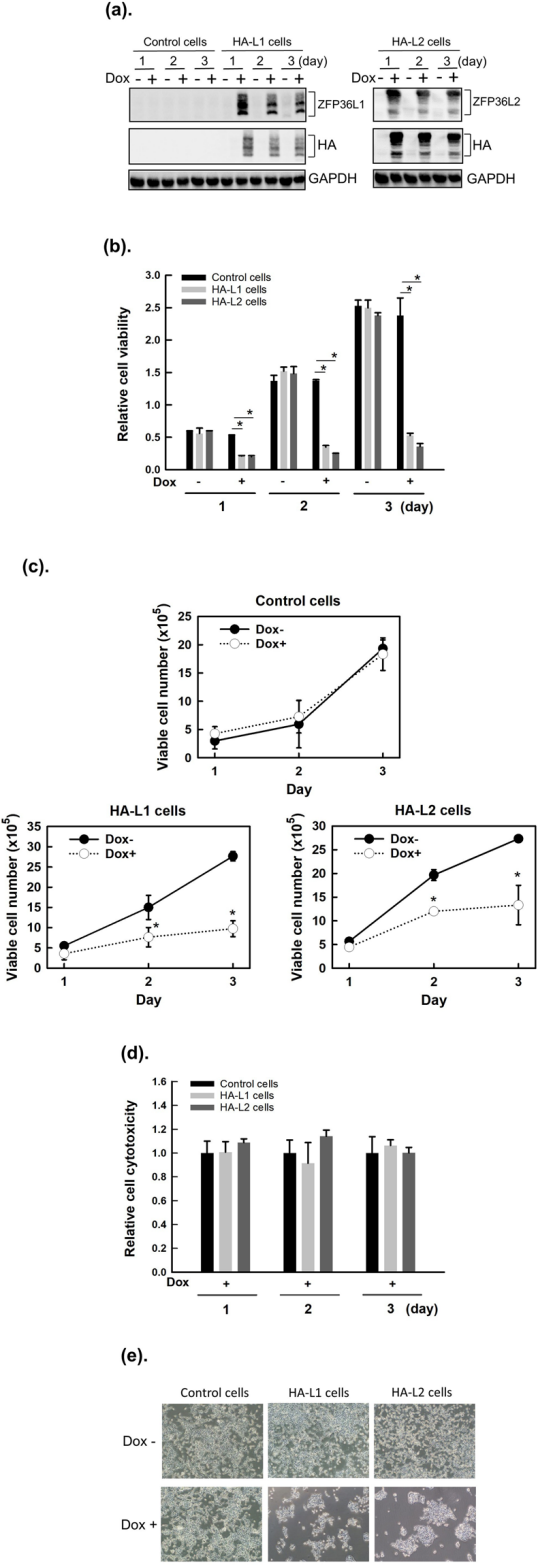
Overexpression of ZFP36L1 and ZFP36L2 decreased the viability of T-REx-293 cells. T-REx-293/pcDNA5-TO (control), T-REx-293/HA-ZFP36L1 (HA-L1), and T-REx-293/HA-ZFP36L2 (HA-L2) cells were treated with 1 μg/mL of doxycycline (Dox) to induce ZFP36L1 or ZFP36L2 expression for 1, 2, and 3 days. (**a**) Total proteins were collected, and the protein expressions of hemagglutinin (HA)-tagged ZFP36L1 or HA-tagged ZFP36L2 were examined using western blot analysis with anti-ZFP36L1, anti-ZFP36L2, or anti-HA antibodies. (**b**) Cell viability was analyzed using an MTT method. Values were normalized to control cells without Dox at day 1 (lane 1). (**c**) The numbers of viable cells were determined by counting the trypan blue-excluding cells in a hemocytometer. (**d**) Cytotoxicity was analyzed using a lactate dehydrogenase cytotoxicity detection kit. (**e**) After 72 h of treatment, the cells were photographed; the representative photographs are shown. Each value is presented as the mean ± SD of three independent experiments. *p < 0.05, compared with control cells.

To examine whether ZFP36L1 or ZFP36L2 could affect cell proliferation, we first used the Tet-On inducible system to overexpress hemagglutinin-(HA-)tagged ZFP36L1 and HA-tagged ZFP36L2 in T-REx-293/HA-ZFP36L1 and T-REx-293/HA-ZFP36L2 cells, respectively. As shown in Fig. 1a, doxycycline (Dox, a more-stable tetracycline analog) treatment induced ZFP36L1 and ZFP36L2 protein expression, which persisted for at least 3 days, using the anti-BRF1/2 (which recognizes both ZFP36L1 and ZFP36L2) antibody, anti-TIS11D (which recognizes ZFP36L2) antibody, and anti-HA antibody. The endogenous ZFP36L1 and ZFP36L2 expression were very low in T-REx-293 cells. Overexpression of ZFP36L1 and ZFP36L2 significantly decreased viable cell numbers at 1, 2, and 3 days after Dox treatment (Fig. 1b and c). To investigate whether the decrease in viable cells was caused by cell death, we used lactate dehydrogenase (LDH) to determine the cytotoxicity of cells. As shown in Fig. 1d, no difference existed in the cytotoxicity of the control cells with T-REx-293/HA-ZFP36L1 cells or the control cells with T-REx-293/HA-ZFP36L2 cells. These results indicate that overexpression of ZFP36L1 or ZFP36L2 can inhibit cell proliferation but does not cause cell death.

### ZFP36L1 and ZFP36L2 induced cell cycle arrest in the G_1_ phase and downregulated the expression of G_1_ phase-related cell cycle molecules

**Figure 2:**
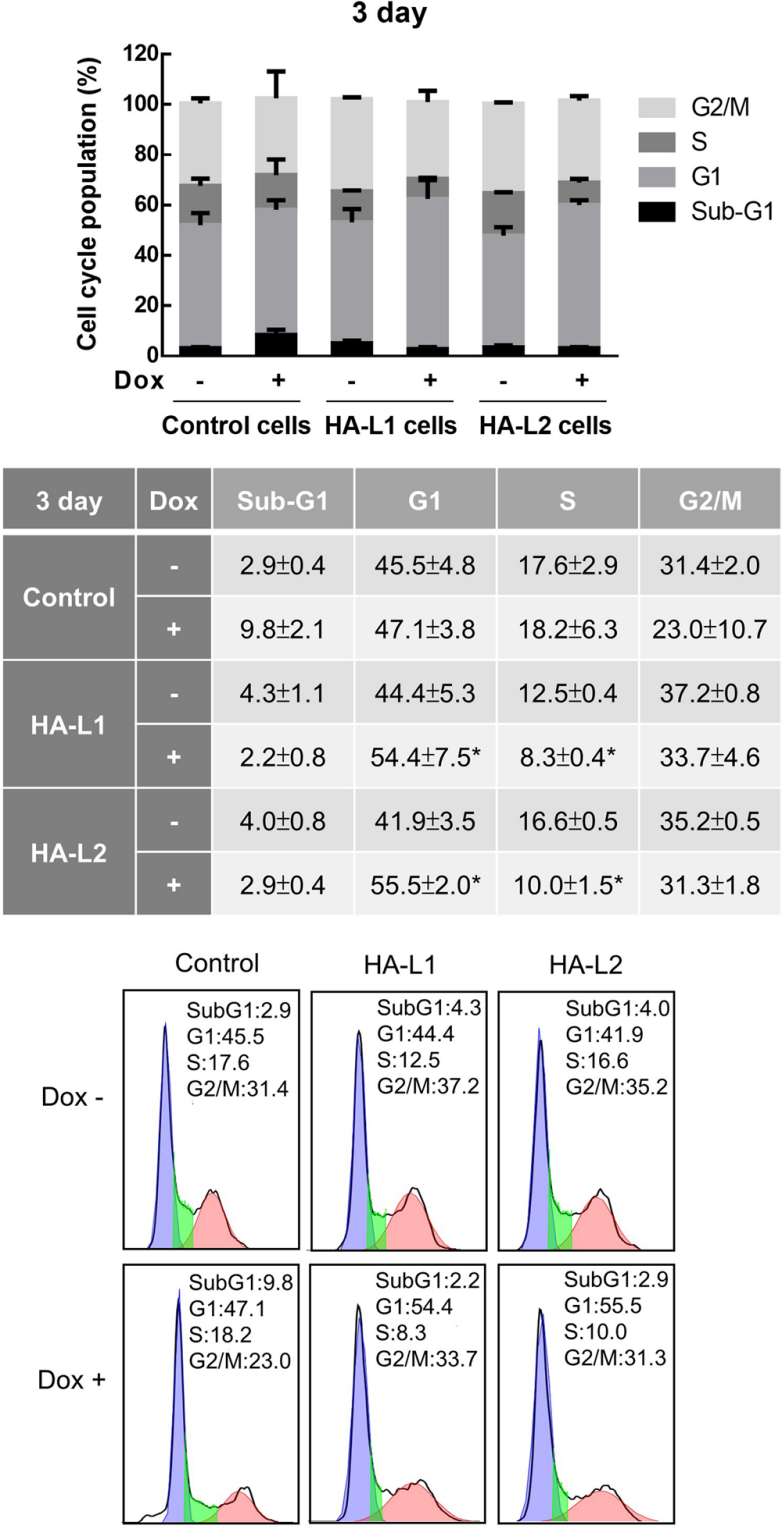
Overexpression of ZFP36L1 and ZFP36L2 induced cell cycle arrest in the G1 phase of T-REx-293 cells. T-REx-293/pcDNA5-TO (control), T-REx-293/HA-ZFP36L1 (HA-L1), and T-REx-293/HA-ZFP36L2 (HA-L2) cells were treated with 1 μg/mL doxycycline (Dox) to induce ZFP36L1 or ZFP36L2 expression for 1, 2, and 3 days; cell cycle profiles were analyzed using flow cytometry. Each value is presented as the mean ± SD of three independent experiments. *p < 0.01, compared with the same cells without Dox treatment.

To further confirm that overexpression of ZFP36L1 or ZFP36L2 did not cause cell death and to understand the effects of ZFP36L1 or ZFP36L2 on cell cycle distribution, we analyzed cell cycle progression by using flow cytometry. As shown in Fig. 2, overexpression of ZFP36L1 or ZFP36L2 suggests cell cycle arrest at the G_1_ phase on day 3 after Dox treatment; furthermore, it blocked cells from entering the S phase. Dox induced overexpression of ZFP36L1 or ZFP36L2 increased the G_1_ phase population from 44.4% to 54.4% and from 41.9% to 55.5%, respectively, on day 3. By contrast, dox induced overexpression of ZFP36L1 or ZFP36L2 decreased S phase population from 12.5% to 8.3% and from 16.6% to 10.0%, respectively. However, the sub-G_1_ phase population did not increase in either T-REx-293/HA-ZFP36L1 or T-REx-293/HA-ZFP36L2 cells treated with Dox.

**Figure 3:**
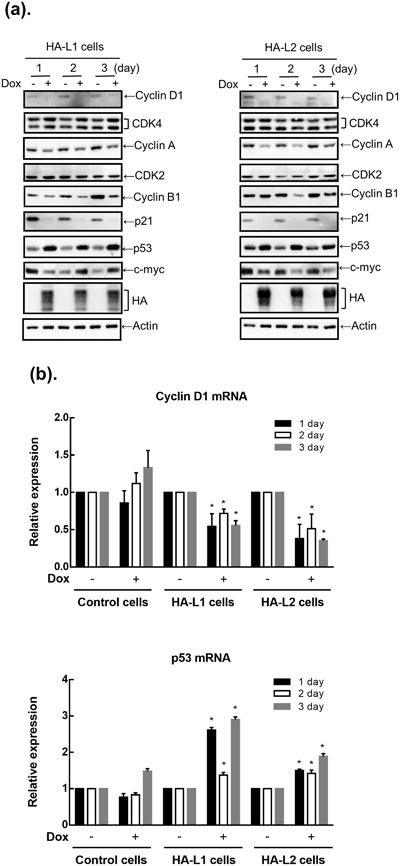
Effects of the overexpression of ZFP36L1 and ZFP36L2 on expressions of cell-cycle-related proteins in T-REx-293 cells. T-REx-293/HA-ZFP36L1 (HA-L1) and T-REx-293/HA-ZFP36L2 (HA-L2) cells were treated with 1 μg/mL doxycycline (Dox) to induce ZFP36L1 or ZFP36L2 expression for 1, 2, and 3 days. (**a**) Total protein was collected, and expressions of cell-cycle-related proteins were detected using western blot analysis. (**b**) Total RNA was collected, and the mRNAs of cyclin D and p53 were detected using a quantitative RT-PCR. Each value is presented as the mean ± SD of three independent experiments. *p < 0.01, compared with the same cells without Dox treatment.

Since ZFP36L1 and ZFP36L2 could arrest the cell cycle in the G_1_ phase, we subsequently examined whether ZFP36L1 or ZFP36L2 could change expressions of G_1_ phase-related cell cycle molecules. Dox induced overexpression of ZFP36L1 or ZFP36L2 indeed downregulated protein expression of cyclin D, cyclin A, and cyclin B1; furthermore, it upregulated p53 protein expression on days 1–3 after Dox treatment (Fig. 3a). However, we cannot exclude the possibility that the changes are due to Dox. On the other hand, no change occurred in the protein expression of Cdk2 or Cdk4 between groups treated with and without Dox. Notably, the p53 downstream target, p21, also decreased in ZFP36L1- or ZFP36L2-overexpressing cells. To examine whether the changes in protein expression were caused by changes in the mRNA expressions of these genes, we detected mRNA expressions of cyclin D and p53 by using a real-time reverse-transcription polymerase chain reaction (RT-PCR). As shown in Fig. 3b, cyclin D mRNA expression decreased and p53 mRNA expression increased on days 1–3 after Dox treatment in both T-REx-293/HA-ZFP36L1 and T-REx-293/HA-ZFP36L2 cells. These results indicate that overexpression of ZFP36L1 or ZFP36L2 can induce cell cycle arrest in the G_1_ phase of T-REx-293 cells, and that underlying molecular mechanisms might be involved in downregulating the mRNA expressions of G_1_ phase-related cyclins and upregulating p53 mRNA expression.

### ZFP36L1 and ZFP36L2 inhibited cell proliferation through a cyclin D-dependent and p53-independent pathway in human colorectal cancer cells

**Figure 4:**
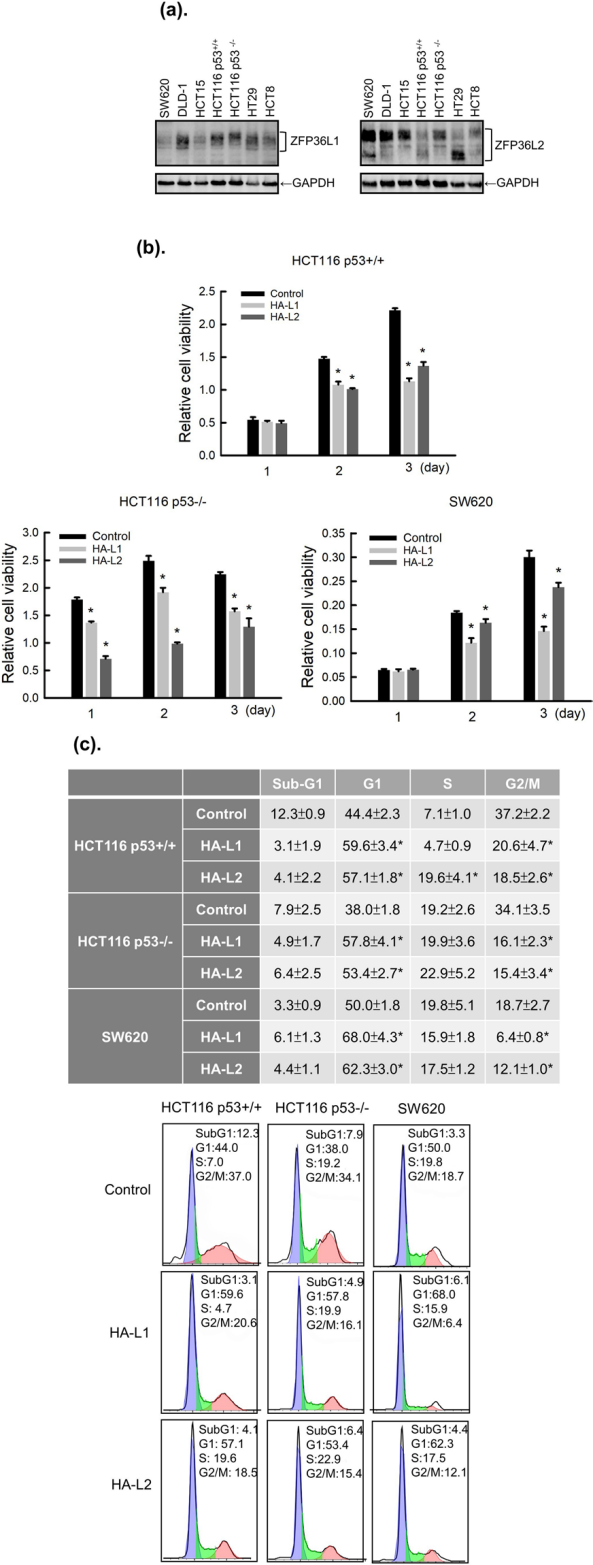
Overexpression of ZFP36L1 and ZFP36L2 decreased cell viability and induced cell cycle arrest in the G1 phase of human colorectal cancer cells. (**a**) Seven human colorectal cancer cell lines, including SW620, DLD-1, HCT15, HCT116 p53^+/+^, HCT116 p53^−/−^, HT29, and HCT8 cells, were cultured for 24 h; the total proteins were collected to detect protein expressions of ZFP36L1 and ZFP36L2 through western blot analysis. (**b,c**) HCT116 p53^+/+^, HCT116 p53^−/−^, and SW620 cells were transduced with a lentiviral expression vector (at a multiplicity of infection of 3) to express enhanced green fluorescent protein (eGFP; control), heme agglutinin (HA)-tagged ZFP36L1 (HA-L1), or HA-tagged ZFP36L2 (HA-L2) protein for 1, 2, and 3 days. (**b**) Cell viability was analyzed using an MTT method. Each value is presented as the mean ± SD of three independent experiments. *p < 0.05, compared with control cells. (**c**) After 72 h of treatment, cell cycle profiles were analyzed using flow cytometry.

**Figure 5:**
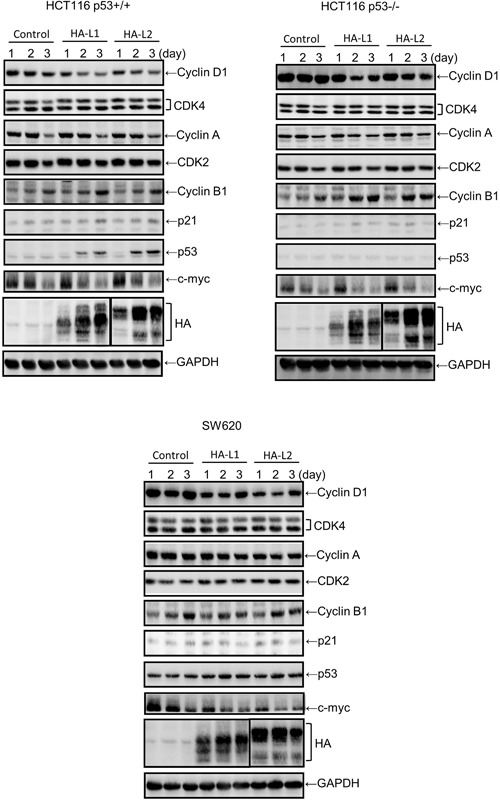
Effects of the overexpression of ZFP36L1 and ZFP36L2 on expressions of cell-cycle-related proteins in human colorectal cancer cells. HCT116 p53^+/+^, HCT116 p53^−/−^, and SW620 cells were transduced with a lentiviral expression vector (at a multiplicity of infection of 3) to express enhanced green fluorescent protein (eGFP; control), hemagglutinin (HA)-tagged ZFP36L1 (HA-L1), or HA-tagged ZFP36L2 (HA-L2) protein for 1, 2, and 3 days. Total proteins were collected, and expressions of cell-cycle-related proteins were detected using western blot analysis.

**Figure 6:**
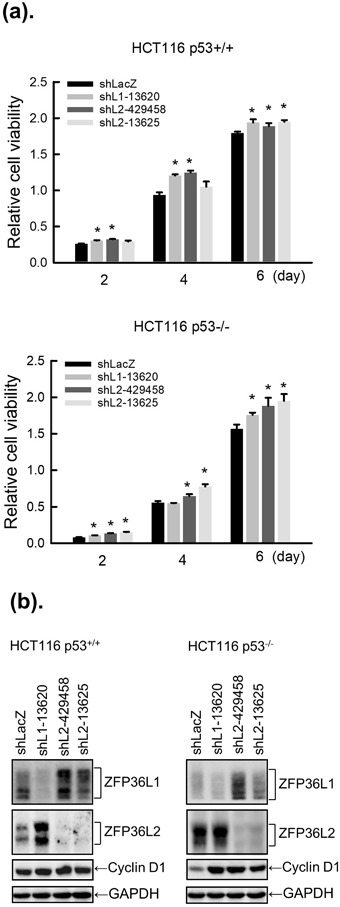
Knockdown of ZFP36L1 or ZFP36L2 increased cell viability and cyclin D expression in human colorectal cancer cells. In HCT116 p53^+/+^ or HCT116 p53^−/−^ cell lines, expression of ZFP36L1 or ZFP36L2 was respectively knocked down by shLacZ (control), shZFP36L1-13620 (shL1-13620), shZFP36L2-429458 (shL2-429458), and shZFP36L2-13625 (shL2-13625) for 2, 4, and 6 days. (**a**) Cell viability was analyzed using an MTT method. Each value is presented as the mean ± SD of three independent experiments. *p < 0.05, compared with control cells. (**b**) After 4 days of treatment, total proteins were collected to detect cyclin D protein expression through western blot analysis.

To confirm the effects of ZFP36L1 and ZFP36L2 on tumor cells, we used seven human colorectal cancer cell lines to screen expressions of ZFP36L1 and ZFP36L2 by using western blot analysis. Regardless of their malignancy, these seven cell lines expressed varying levels of endogenous ZFP36L1 and ZFP36L2 (Fig. 4a). Relevant studies have demonstrated that ZFP36 can regulate cell proliferation through the p53 molecule. To examine whether ZFP36L1 and ZFP36L2 regulate cell proliferation through p53, we selected HCT116 p53^+/+^, HCT116 p53^−/−^, and SW620 cells for which the p53 statuses are wild type (WT), null, and mutated, respectively. First, the ZFP36L1 or ZFP36L2 gene was transduced into these three cell lines by a lentivirus. Forced expression of ZFP36L1 or ZFP36L2 had significantly decreased proliferation of these three types of cells 2 days after lentivirus transduction (Fig. 4b). Subsequently, we examined whether forced expression of ZFP36L1 or ZFP36L2 could also result in cell cycle arrest in the G_1_ phase in the three cell lines. Similar to T-REx-293 cells, forced expression of ZFP36L1 or ZFP36L2 also suggests G_1_ phase arrest in the three cell lines (Fig. 4c). With further detection of G_1_ phase-related molecule expressions, we found that forced expression of ZFP36L1 or ZFP36L2 markedly reduced cyclin D protein expression but did not change expressions of other molecules, including cyclin A, Cdk2, or Cdk4, in these three types of colorectal cancer cell (Fig. 5). By contrast, forced expression of ZFP36L1 or ZFP36L2 seemed to increase cyclin B1 expression in HCT116 p53^+/+^ and HCT116 p53^−/−^ cells and induce p53 and p21 expressions in HCT116 p53^+/+^ cells. We subsequently examined whether knockdown of ZFP36L1 or ZFP36L2 could increase the proliferation of HCT116 p53^+/+^ and HCT116 p53^−/−^ cells. Knockdown of ZFP36L1 or ZFP36L2 by lentivirus-delivered shZFP36L1-13620, shZFP36L2-429458, or shZFP36L2-13625 significantly increased the cell number and cyclin D protein expression (Fig. 6). Although this increase in cyclin D1 for HCT116 p53^+/+^ cells is minor, the increase of between 1.5 and 2.1 from densitometry is significant. These results suggest that ZFP36L1 and ZFP36L2 can inhibit cell proliferation in a cyclin D-dependent and p53-independent manner in human colorectal cancer cells.

### Zinc finger domains of ZFP36L1 and ZFP36L2 are required for downregulation of cyclin D expression

**Figure 7:**
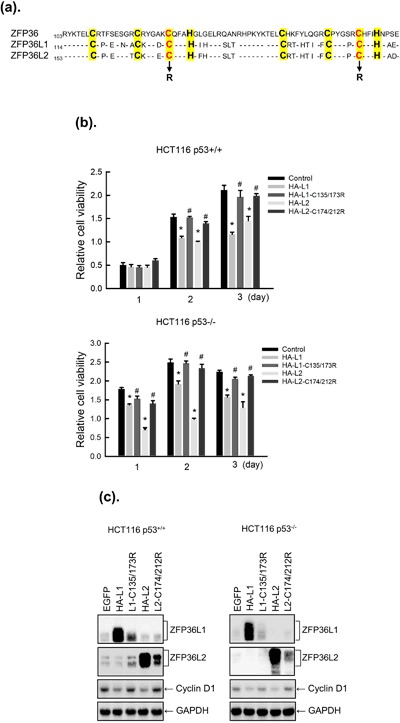
Mutation in the tandem zinc finger (TZF) domain of ZFP36L1 and ZFP36L2 prevented the inhibitory effects on the viability and cyclin D expression of human colorectal cancer cell lines. (**a**) Sequence of the TZF domains, each of which contained three conserved cysteine residues and one conserved histidine residue (marked in yellow). The mutation sites of ZFP36L1-C135/173R and ZFP36L2-C174/212R contracts are shown. (**b**,**c**). HCT116 p53^+/+^ and HCT116 p53^−/−^ cells were transduced with a lentiviral expression vector (at a multiplicity of infection of 3) to express enhanced green fluorescent protein (eGFP; control), hemagglutinin (HA)-tagged ZFP36L1 (HA-L1), HA-tagged ZFP36L1-C135/173R (HA-L1-C135/173R), HA-tagged ZFP36L2 (HA-L2), or HA-tagged ZFP36L2-C174/212R (HA-L2-C174/212R) for 1, 2, and 3 days. (**b**) Cell viability was analyzed using an MTT method. Each value is presented as the mean ± SD of three independent experiments. *p < 0.05, compared with control cells. ^#^*p* < 0.05, compared with individual wild-type cells. (**c**) After 2 days of treatment, the total proteins were collected to detect cyclin D protein expression through western blot analysis.

A previous study found that ZFP36L1 and ZFP36L2 have two TZF domains, which are critical for binding the AU-rich element of mRNA 3′UTR and lead to mRNA degradation^18^. Thus, we investigated whether these two TZF domains affected cyclin D expression and cell proliferation in HCT116 p53^+/+^ and HCT116 p53^−/−^ cells. The cysteine residue of each TZF domain was replaced with arginine to abolish their mRNA-binding activities and generate the ZFP36L1-C135/173R and ZFP36L2-C174/212R mutant plasmids (Fig. 7a). HCT116 p53^+/+^ and HCT116 p53^−/−^ cells were transduced with lentiviruses expressing HA-ZFP36L1, HA-ZFP36L1-C135/173R, HA-ZFP36L2, and HA-ZFP36L2-C174/212R; subsequently, the viable cell number and cyclin D expression were determined (Fig. 7b and c). The WT ZFP36L1 and ZFP36L2—but not ZFP36L1-C135/173R or ZFP36L2-C174/212R mutant—inhibited cell proliferation and cyclin D expression, which suggests that the TZF domains of ZFP36L1 and ZFP36L2 are required for their antiproliferative activity and cyclin D degradation. However, we cannot exclude the possibility that the ZFP36L1-C135/173R and ZFP36L2-C174/212R did not inhibit proliferation or cyclin D expression were because they were expressed at a lower level than WT.

## Discussion

In this study, we found that overexpression of ZFP36L1 or ZFP36L2 inhibited cell proliferation but did not cause the death of T-REx-293/HA-ZFP36L1 or T-REx-293/HA-ZFP36L2 cells. Further analysis found that overexpression of ZFP36L1 or ZFP36L2 inhibited expressions of cell-cycle-related molecules, including cyclin B, cyclin D, and cyclin A proteins; furthermore, it increased p53 expression but reduced expression of the p53 downstream target gene, p21. Subsequently, we overexpressed ZFP36L1 or ZFP36L2 in human colorectal cancer HCT116 p53^+/+^, HCT116 p53^−/−^, and SW620 cells, and discovered that proliferation of these cells was inhibited regardless of their p53 status. Similar to T-REx-293 cells, expression of cyclin D protein in these cells also decreased, but expressions of cyclin B and cyclin A proteins were unchanged. Knockdown of ZFP36L1 or ZFP36L2 increased proliferation and cyclin D expression in these cells. Moreover, mutation of the TZF domains of ZFP36L1 or ZFP36L2 appears to abolish their inhibitory abilities on cell proliferation and cyclin D expression.

Relevant studies have shown that overexpression of ZFP36 inhibited the growth of MDA-MB-231 breast cancer cells but did not cause apoptosis^38^; however, overexpression of ZFP36L2 also inhibited cell growth but activated apoptosis (e.g., activation of caspase-3 and poly(ADP ribose) polymerase cleavage) in HeLa cells^39^. In this study, overexpression of ZFP36L1 or ZFP36L2 did not cause apoptosis in T-REx-293 cells or in human colorectal cancer HCT116 p53^+/+^, HCT116 p53^−/−^, and SW620 cells. These results suggest that the induction of apoptosis by the ZFP36 family is dependent on cell or tissue type. When inducible knockout mice were used, double knockout of ZFP36L1 and ZFP36L2 in the thymus led to the development of acute T lymphoblastic leukemia; however, knockdown of either ZFP36L1 or ZFP36L2 alone did not cause the disease to develop^40^. In this study, we also found that knockdown of ZFP36L1 increased ZFP36L2 expression, whereas knockdown of ZFP36L2 increased ZFP36L1 expression. However, such compensation did not restore its inhibitory effect on cell proliferation and cyclin D expression (Fig. 6b). These results suggest that ZFP36L1 and ZFP36L2 might compensate for each other in gene expression and certain biological activities.

A critical tumor suppressor gene, p53 is typically found with mutations or deletions in many types of human cancers^41^. It can inhibit cell cycle progression by inducing p21 and subsequently inhibiting Cdk activity. Dox induced overexpression of ZFP36L1 or ZPF36L2 increased p53 expression; however, p21 was not induced by p53 (Fig. 3a) because p21 mRNA is also an ARE-containing target of ZFP36L1, and ZFP36L1 and ZFP36L2 deficiency increased p21 mRNA level^27,42^. Studies have shown that forced expression of ZFP36L2 increased p53 expression in HeLa cells^39^; however, overexpression of p53 also increased ZFP36L2 expression in human colorectal cancer DLD1 cells^43^. Those findings indicated that the ZFP36 family and p53 can regulate each other. In addition, ZFP36 expression was induced by Dox in a p53-dependent manner in human ovarian cancer cells, and the induction of p53 may have contributed to ZFP36-mediated apoptosis^44^. In this study, overexpression of ZFP36L1 or ZFP36L2 inhibited proliferation of three human cell lines with various p53 statuses, including HCT116 p53^+/+^ (WT p53), HCT116 p53^−/−^ (null p53), and SW620 (p53 mutation), indicating that ZFP36L1 or ZFP36L2 inhibited cell proliferation in a p53-independent manner.

ZFP36 family proteins were identified as AU-rich element-binding proteins (ARE-BPs) that can regulate ARE-containing mRNA stability and translational activity. One study showed that the TZF domain of the CCCH-type zinc finger protein is crucial for recognizing and binding to AREs and in mRNA degradation. Defects in ZFP36 TZF increased the stability of TNF-α mRNA and subsequently caused inflammation through the overproduction of TNF-α^45^; therefore, defects in ZFP36 TZF can lead to a systemic inflammatory syndrome and autoimmunity in mice. ZFP36 TZF mutations also increased the growth of breast cancer cells^46^. In addition, mutated ZFP36L1 (C135/173R) in the TZF domain lost its ability to bind to Notch1 mRNA in T lymphocytic leukemia cells, and led to overexpression of the Notch1 gene. In this study, we suggest that mutated ZFP36L1 (C135/173R) and ZFP36L2 (C174/212R) were unable to inhibit the expression of cyclin D and cell proliferation, suggesting that the TZF domains of ZFP36L1 and ZFP36L2 are critical for the inhibition of cyclin D expression and cell proliferation. These results suggest that ZFP36L1 or ZFP36L2 can inhibit cell proliferation through downregulation of cyclin D expression, resulting in cell cycle arrest in the G_1_ phase, and furthermore, the TZF domains of ZFP36L1 or ZFP36L2 might be crucial in the downregulation of cyclin D.

## Materials and Methods

### Cell lines and antibodies

Human embryonic kidney HEK293 cells and tetracycline (Tet)-regulated expression embryonic kidney T-REx-293/pcDNA5-TO (control cells), T-REx-293/HA-ZFP36L1, and T-REx-293/HA-ZFP36L2 cell lines were kindly provided by Dr. Yi-Ling Lin (Institute of Biomedical Sciences, Academia Sinica, Taipei, Taiwan) and grown in Dulbecco’s modified Eagle’s medium containing 10% fetal bovine serum. Human colon adenocarcinoma SW620, DLD-1, HCT15, HCT116 p53^+/+^, HT29, and HCT8 cells were obtained from the Food Industry Research and Development Institute (FIRDI, Hsinchu, Taiwan) and grown according to FIRDI suggestions. HCT116 p53^−/−^ was kindly provided by Dr. Zee-Fen Chang (Institute of Molecular Medicine, School of Medicine, National Taiwan University, Taipei, Taiwan). Rabbit polyclonal anti-BRF1/2, mouse monoclonal anti-cyclin D, rabbit polyclonal anti-CDK2, rabbit polyclonal anti-c-Myc, and mouse monoclonal anti-p53 antibodies were purchased from Cell Signaling Technology (Danvers, MA, USA); rabbit polyclonal anti-cyclin B1, rabbit polyclonal anti-cyclin A2, and rabbit polyclonal anti-GAPDH antibodies were purchased from GeneTex (Irvine, CA, USA); and rabbit polyclonal anti-CDK4 and mouse monoclonal anti-TIS11D antibodies were purchased from Santa Cruz Biotechnology (Dallas, TX, USA). Mouse monoclonal anti-hemagglutinin (HA) (Covance, Princeton, NJ, USA), mouse monoclonal anti-p21 (BD Biosciences, Franklin Lakes, NJ, USA), and mouse monoclonal anti-actin (Novus Biologicals, Littleton, CO, USA) antibodies were also used in this study.

### Plasmids and lentivirus

Complementary (c)DNA of human ZFP36L1 and ZFP36L2 with an N-terminal HA-tag were subcloned into a lentiviral expression construct (pSIN) named pSIN-HA-ZFP36L1 (WT) and pSIN-HA-ZFP36L2 (WT), respectively. The HA-tagged mutant constructs of ZFP36L1-C135/173R and ZFP36L2-C174/212R were generated from the human ZFP36L1 and ZFP36L2 WT constructs, respectively. The lentiviral vector plasmid pSIN-EGFP, which contained an enhanced green fluorescent protein (eGFP) gene, served as the control plasmid. These five plasmids were kindly provided by Dr. Yi-Ling Lin (Institute of Biomedical Sciences, Academia Sinica, Taipei, Taiwan).

To knock down the endogenous ZFP36L1 or ZFP36L2, we used shZFP36L1-13620 (clone ID: shRNA TRCN0000013620), shZFP36L2-429458 (clone ID: shRNA TRCN00000429458), and shZFP36L2-13625 (clone ID: shRNA TRCN0000013625) lentiviral particles (National Core Facility for Manipulation of Gene Function by RNAi, miRNA, miRNA Sponges, and CRISPR/Genomic Research Center, Academia Sinica, supported by the National Core Facility Program for Biotechnology Grants of MOST (MOST 104-2319-B-001-001-)). The following target sequences were constructed into the pLKO.1 shRNA cloning vector: ZFP36L1-13620, 5′-GTAACAAGATGCTCAACTATA-3′; ZFP36L2-429458, 5′-ATCAACTCCACGCGCTACAAG-3′; and ZFP36L2-13625, 5′-CTTCTTGTGCAAGACAGAGAA-3′. A short hairpin (sh)RNA vector against β-galactosidase (pLKO.1-shLacZ) was used as a negative control for knockdown validation. The generation and titer of lentiviruses were based on a previously described method^47^.

### Western blot analysis

Total cellular proteins (10–30 μg) were extracted and chromatographed with sodium dodecyl sulfate polyacrylamide gel electrophoresis (SDS-PAGE) as described previously^48^. After being transferred to a polyvinylidene difluoride (PVDF) membrane, the membrane was blocked in 1% bovine serum albumin or 10% nonfat milk for 1 h before incubation with specific primary antibodies. Secondary antibodies conjugated to horseradish peroxidase (HRP) were used to detect antigen–antibody complexes by using an enhanced chemiluminescence kit (Thermo Fisher Scientific Taiwan, Taipei, Taiwan) in an ImageQuant LAS 4000 Biomolecular Imager (GE Healthcare Life Sciences, Marlborough, MA).

### Real-time RT-PCR

Total RNA was isolated with a Trizol RNA extraction reagent^49^, and cDNA was synthesized using a GoScript Reverse Transcription System (Promega, Madison, WI) according to the manufacturer’s instructions. Two microliters of cDNA was used to perform the real-time PCR on an Applied Biosystems StepOne Real-Time PCR System with Applied Biosystems SYBR Green PCR Master Mix according to the manufacturer’s instructions. The total 20-μL volume contained 1 μg of cDNA, 0.2 μM of each primer, and 10 μL of SYBR Green PCR Master Mix. The following oligonucleotide primers were used: cyclin D, forward 5′-AGGAACAGAAGTGCGAGGAGG-3′ and reverse 5′-GGATGGAGTTGTCGGTGTAGATG-3′; p53, forward 5′-CCCCTCCTGGCCCCTGTCATCTTC-3′ and reverse 5′-GCAGCGCCTCACAACCTCCGTCAT-3′; and GAPDH, forward 5′-AGCCACATCGCTCAGACAC-3′ and reverse 5′-GCCCAATACGACCAAATCC-3′. The conditions of the real-time PCR were: 95 °C preincubation for 20 s; 40 cycles of 95 °C for 20 s, 60 °C for 30 s, and 72 °C for 30 s for amplification; and 95 °C for 15 s, 60 °C for 1 min, and 95 °C for 15 s for a melting curve analysis. Expression levels of each messenger mRNA were calculated using the comparative Ct method (ΔΔCt formula) and normalized to the GAPDH mRNA level^50^.

### Cell viability assay

Cells were washed with phosphate-buffered saline (PBS) twice, and incubated with 200 μL of culture medium and 50 μL of a 3-(4,5-dimethyl-2-thiazolyl)-2,5 diphenyl-2H-tetrazolium, thiazolyl blue tetrazolium bromide (MTT, 5 mg/ml; Sigma Chemical, St. Louis, MO) solution at 37 °C for 30 min in the dark. After the MTT solution was removed, 200 μL of DMSO was added to each cell, and the absorbance at 540 nm was measured with an enzyme-linked immunosorbent assay reader^51^.

### Flow cytometry

The population of the cell cycle was analyzed using flow cytometry as previously described^52^. Briefly, cells were trypsinized, washed twice with PBS, and fixed in 75% ethanol for 1 h at −20 °C. The fixed cells were then washed with PBS, incubated with 0.5 mL of PBS containing 0.05% RNase and 0.5% Triton X-100 for 30 min at 37 °C, and stained with propidium iodide. The stained cells were analyzed with FACScan flow cytometry using CellQuest 3.3 analytical software (Becton Dickinson, San Jose, CA).

### Statistical analysis

Statistical analyses were performed using a one-way Student’s *t*-test in SigmaPlot 13.0; differences were considered significant at *p* < 0.05.
