# Author Correction: ZFP36L1 and ZFP36L2 inhibit cell proliferation in a cyclin D-dependent and p53-independent manner

**DOI:** 10.1038/s41598-019-53894-9

**Published:** 2019-11-20

**Authors:** Fat-Moon Suk, Chi-Ching Chang, Ren-Jye Lin, Shyr-Yi Lin, Shih-Chen Liu, Chia-Feng Jau, Yu-Chih Liang

**Affiliations:** 1 Division of Gastroenterology, Department of Internal Medicine, Wan Fang Hospital, Taipei Medical University, Taipei, Taiwan; 2 Division of Allergy, Immunology and Rheumatology, Department of Internal Medicine, School of Medicine, College of Medicine, Taipei Medical University, Taipei, Taiwan; 3 Division of Rheumatology, Immunology and Allergy, Taipei Medical University Hospital, Taipei, Taiwan; 4 School of Medical Laboratory Science and Biotechnology, College of Medical Science and Technology, Taipei Medical University, Taipei, Taiwan; 5 Department of Primary Care Medicine, Taipei Medical University Hospital, Taipei, Taiwan; 6 Department of General Medicine, School of Medicine, College of Medicine, Taipei Medical University, Taipei, Taiwan; 7 Traditional Herbal Medicine Research Center, Taipei Medical University Hospital, Taipei, Taiwan; 8 Ph.D. Program in Medical Biotechnology, College of Medical Science and Technology, Taipei Medical University, Taipei, Taiwan

Correction to: *Scientific Reports* 10.1038/s41598-018-21160-z, published online 09 February 2018

The original version of this Article contained an error in Affiliation 2, which was incorrectly given as ‘Department of Internal Medicine, School of Medicine, College of Medicine, Taipei Medical University, Taipei, Taiwan’. The correct affiliation is listed below:

Division of Allergy, Immunology and Rheumatology, Department of Internal Medicine, School of Medicine, College of Medicine, Taipei Medical University, Taipei, Taiwan.

This error has now been corrected in the HTML and PDF versions of the Article.

